# A Critical Appraisal of the Congress of Neurological Surgeons Evidence-Based Guidelines on the Evaluation and Treatment of Patients With Thoracolumbar Spine Trauma

**DOI:** 10.7759/cureus.58641

**Published:** 2024-04-20

**Authors:** Navnit S Makaram, Ning Liang, Sizhan Wu, Simon B Roberts, James Ngwayi, Patrick Statham, Daniel E Porter

**Affiliations:** 1 Department of Orthopaedics and Traumatology, Royal Infirmary of Edinburgh, Edinburgh, GBR; 2 Department of Orthopaedics, Beijing Huaxin Hospital, School of Clinical Medicine, Tsinghua University, Beijing, CHN; 3 Department of Orthopaedics, School of Clinical Medicine, Tsinghua University, Beijing, CHN; 4 Department of Orthopaedics, Leeds General Infirmary, Leeds, GBR; 5 Department of Neurosurgery, Western General Hospital, Edinburgh, GBR

**Keywords:** lumbar, thoracic, trauma, congress of neurological surgeons, clinical practice guideline, agree ii

## Abstract

Background and objective

Thoracolumbar spine trauma (TST) is frequently associated with spinal cord injury and other soft tissue and bony injuries. The management of such injuries requires an evidence-based approach. This study used the Appraisal of Guidelines for Research and Evaluation (AGREE) II instrument to assess the methodological quality of clinical guidelines for the management of TST published by the Congress of Neurological Surgeons (CNS).

Methods

All clinical guidelines on TST published by CNS until 2020 were assessed. Five appraisers from three international centers evaluated the quality of eligible clinical guidelines by using AGREE II. Mean AGREE II scores for each domain were determined. In higher-quality domains, the scores for individual items were analyzed.

Results

A total of 12 guidelines published by CNS on TST were assessed. Mean scores for all six domains were as follows: Scope and Purpose (75.2%), Stakeholder Involvement (45.4%), Rigor of Development (57.0%), Clarity of Presentation (58.7%), Applicability (16.9%), and Editorial Independence (64.1%). The mean score for the overall quality of all CNS guidelines was 52.9% [95% confidence interval (CI): 52.2-53.5%]. The overall agreement among appraisers was excellent [intra-class correlation coefficients (ICCs) for each guideline ranged from 0.903 to 0.963].

Conclusions

CNS guidelines for the management of TST demonstrated acceptable quality across most domains; however, the domains of Applicability and Stakeholder Involvement could be further improved in future guideline updates. The assessors concluded that all guidelines could still be recommended for clinical practice with or without modifications.

## Introduction

Traumatic injuries of the thoracolumbar spine are reported in around 7% of all blunt trauma patients and account for 50-90% of traumatic spinal fractures in North America every year [1-5]. Furthermore, up to 25% of patients affected by traumatic thoracolumbar fractures suffer from an associated spinal cord injury [2,4]. The rationale for evaluating injuries of the thoracolumbar spine separately from the cervical spine lies in the disparate anatomic, physiologic, and epidemiologic characteristics of this fracture group [2]. Compared to the cervical spine, thoracolumbar fractures are more common and evaluated using a separate classification system. However, universally accepted and internationally applicable guidelines for the management of thoracolumbar spine trauma (TST) have not been published yet, due to both a lack of consensus on a variety of aspects of TST management and a dearth of available high-quality evidence. Furthermore, the critical appraisal of such evidence and their incorporation into clinical practice guidelines (CPG) has not previously been clearly described; no prior TST guidelines have directed the reader to the published evidence showing the reasoning for guideline formulation or described the quality of such evidence.

The American Association of Neurological Surgeons (AANS) is a scientific and educational association and is one of the largest such bodies in the world dedicated to neurosurgery. It comprises more than 12,000 members worldwide. The American Association of Neurological Surgeons/Congress of Neurological Surgeons has previously published widely accepted guidelines on traumatic brain injury [6], metastatic brain tumors [7], and spinal cord injury [8] among others. The AANS/CNS published guidelines on the evaluation and treatment of TST in 2018, aiming to provide a widely accepted and applicable set of guidelines with clear reference to methodology, evidence base, and quality of evidence [9]. These were laudable aims and were critically required at the time of their publication.

The development of established clinical guidelines must involve the use of rigorous methodological steps to minimize the risk of bias [10,11]. It is equally crucial that guidelines should undergo external peer appraisal using a widely established and recognized tool. There are several assessment tools developed to evaluate the methodological quality of clinical guidelines [12]. Among them, the Appraisal of Guidelines for Research and Evaluation II (AGREE II) instrument is widely used, and it is recommended in the WHO Handbook for Guideline Development [13,14]. The AGREE II tool benchmarks methodological strategies for the development of new guidelines [15,16].

The available evidence suggests that clinicians choose to read and follow guidelines written by trusted national and international professional bodies. A recent survey of Chinese spine surgeons revealed that 86% of the participants understood the Chinese Association of Orthopaedic Surgeons guidelines, and 44% understood AO Spine guidelines [17]. In addition, some organizations specifically use the AGREE II methodology to internally audit guideline quality during their development phase [18]. However, internal audits are susceptible to the risk of bias, highlighting the significance of external appraisals to provide an independent quality evaluation.

While the AGREE II tool has been used to assess several sets of guidelines in orthopedics, neurological injury, and neurorehabilitation [19-22], there is no such evaluation of guidelines specifically regarding the management of TST based on AGREE II. Furthermore, the quality of CNS guidelines on TST has never previously been subject to formal external critical appraisal using an independently validated tool such as AGREE II. In light of this, this study primarily aimed to assess the quality of CNS-derived CPGs on TST and related pathologies according to the AGREE II criteria and to determine specific areas that could be improved upon in future iterations of the guidelines.

## Materials and methods

Guideline identification

The search for TST guidelines was carried out by two researchers (NSM and NL) using the organization’s website [23] until January 1, 2020. The key search terms used were as follows: “thoracic spine”, “lumbar spine”, or “thoracolumbar” and "guideline". Both a keyword search and a manual search were performed, adhering to The Preferred Reporting Items for Systematic Reviews and Meta-Analysis (PRISMA) guidelines [24]. The inclusion criteria were as follows: 1) literature relating to TST or related complications; 2) literature that met the guidelines standard of the National Guidelines Clearinghouse [25].

AGREE II instrument

The AGREE II tool contains 23 items in six domains and two overall assessment items. The six domains are as follows: Scope and Purpose (three items), Stakeholder Involvement (three items), Rigor of Development (eight items), Clarity of Presentation (three items), Applicability (4 items), and Editorial Independence (two items) [13,14]. Each item is rated on a 7-point scale [ranging from 1 (strongly disagree) to 7 (strongly agree)] [13,14]. The two overall assessment items were as follows: 1) ‘rate the overall quality of the guideline’ scored on a 7-point scale’, and 2) ‘would recommend the guideline for use’ scored as one of three responses: ‘yes’, ‘yes with modifications’ and ‘no’ [13,14].

Ethics statement

This study is an evaluation of existing literature and did not involve any human subjects; hence, it is not subject to ethics committee evaluation.

Evaluation of the guidelines

Each guideline was assessed by a panel of five appraisers comprising four orthopedic surgeons with interests in spine surgery, and a neurosurgeon with a special interest in spine surgery (NSM, NL, SW, PS, and DP). All were either native English speakers or able to evaluate medical English literature proficiently. A minimum of four appraisers rated each guideline; the reliability of the AGREE II tool had been previously found to be greater when four assessors were involved than with two or three [26]. The appraisers each completed the online training course required by AGREE II before undertaking the guideline assessment [27].

Statistical analysis

The calculation method to obtain scores for each domain involved summing up the scores of all items in the domain and recording this as a percentage of the maximum possible score for the domain [16]. Mean values [and 95% confidence intervals (CI)] for all raters were calculated. Although domain scores can be used to compare different guidelines and to help determine whether guidelines should be recommended, this is not determined by the AGREE II tool and is instead determined by the user [13,14]. In line with existing literature evaluating guidelines using the AGREE II methodology, the domain score criteria we used were as follows: <40%: very low quality, 40%-59%: low quality, 60%-79%: acceptable quality, and ≥80%: good quality [28,29].

Scores obtained for individual items within domains were calculated using the same method as for domain scores. Given that the purpose of the AGREE scale is to encourage best practice, we took the view that the domains that failed to reach the "acceptable" or "good" quality threshold should not be the focus of our assessment. Hence, although all CNS guideline domain quality scores are presented, item scores are only displayed and discussed for domains that reach “acceptable” quality.

Inter-rater reliability of domain scores was assessed using the intra-class correlation coefficient (ICC). Based on the 95% CI of the ICC estimate, ICC values less than 0.5, those between 0.5 and 0.75, between 0.75 and 0.9, and greater than 0.90 correspond to poor, moderate, good, and excellent reliability, respectively [20].

## Results

A total of 12 guidelines fulfilled the inclusion criteria (Table 1). These were evaluated by five appraisers.

**Table 1:** CNS guidelines on TST CNS: the Congress of Neurological Surgeons; TST: thoracolumbar spine trauma

Title	Number
Congress of Neurological Surgeons Systematic Review and Evidence-Based Guidelines on the Evaluation and Treatment of Patients with Thoracolumbar Spine Trauma: Classification of Injury	CNS-01
Congress of Neurological Surgeons Systematic Review and Evidence-Based Guidelines on the Evaluation and Treatment of Patients with Thoracolumbar Spine Trauma: Executive Summary	CNS-02
Congress of Neurological Surgeons Systematic Review and Evidence-Based Guidelines on the Evaluation and Treatment of Patients with Thoracolumbar Spine Trauma: Hemodynamic Management	CNS-03
Congress of Neurological Surgeons Systematic Review and Evidence-Based Guidelines on the Evaluation and Treatment of Patients with Thoracolumbar Spine Trauma: Neurologic Assessment	CNS-04
Congress of Neurological Surgeons Systematic Review and Evidence-Based Guidelines on the Evaluation and Treatment of Patients with Thoracolumbar Spine Trauma: Nonoperative Care	CNS-05
Congress of Neurological Surgeons Systematic Review and Evidence-Based Guidelines on the Evaluation and Treatment of Patients with Thoracolumbar Spine Trauma: Novel Surgical Strategies	CNS-06
Congress of Neurological Surgeons Systematic Review and Evidence-Based Guidelines on the Evaluation and Treatment of Patients with Thoracolumbar Spine Trauma: Operative Versus Nonoperative Treatment	CNS-07
Congress of Neurological Surgeons Systematic Review and Evidence-Based Guidelines on the Evaluation and Treatment of Patients with Thoracolumbar Spine Trauma: Pharmacological Treatment	CNS-08
Congress of Neurological Surgeons Systematic Review and Evidence-Based Guidelines on the Evaluation and Treatment of Patients with Thoracolumbar Spine Trauma: Prophylaxis and Treatment of Thromboembolic Events	CNS-09
Congress of Neurological Surgeons Systematic Review and Evidence-Based Guidelines on the Evaluation and Treatment of Patients with Thoracolumbar Spine Trauma: Radiological Evaluation	CNS-10
Congress of Neurological Surgeons Systematic Review and Evidence-Based Guidelines on the Evaluation and Treatment of Patients with Thoracolumbar Spine Trauma: Surgical Approaches	CNS-11
Congress of Neurological Surgeons Systematic Review and Evidence-Based Guidelines on the Evaluation and Treatment of Patients with Thoracolumbar Spine Trauma: Timing of Surgical Intervention	CNS-12

The scores of each domain are shown in Table 2, including the overall 95% CI for all domains of included guidelines. All TST guidelines were categorized as having "acceptable" or "good" quality in the domain of Scope and Purpose, with 11 achieving this level of quality for Editorial Independence. Five guidelines achieved this level of quality in the domain of Clarity of Presentation; however, only two guidelines achieved "acceptable" or "good" quality in the domain of Rigor of Development, and no guideline achieved this level of quality for the domains of Stakeholder Involvement or Applicability.

**Table 2:** AGREE II domain scores for each CNS guideline on TST AGREE II: the Appraisal of Guidelines for Research and Evaluation II; CI: confidence interval; CNS: the Congress of Neurological Surgeons; mod: modifications; TST: thoracolumbar spine trauma

Number of guidelines	Domain 1: Scope and Purpose (%)	Domain 2: Stakeholder Involvement (%)	Domain 3: Rigor of Development (%)	Domain 4: Clarity of Presentation (%)	Domain 5: Applicability (%)	Domain 6: Editorial Independence (%)	The overall mean score across all domains	First global rating (personal score) (%)	Second global rating ("I would recommend?")
CNS-01	77.8	44.4	53.1	54.2	18.8	77.1	54.2	50.0	Yes with mod
CNS-02	77.8	48.6	57.8	75.0	18.8	58.3	56.1	57.1	Yes
CNS-03	70.8	44.4	51.6	45.8	21.9	64.6	49.9	50.0	Yes with mod
CNS-04	69.4	43.1	53.1	54.2	17.7	66.7	50.7	50.0	Yes with mod
CNS -05	70.8	41.7	51.0	52.8	14.6	66.7	49.6	50.0	Yes with mod
CNS -06	80.6	45.8	61.5	65.3	17.7	62.5	55.6	53.6	Yes with mod
CNS -07	76.4	47.2	57.8	55.6	16.7	60.4	52.4	53.6	Yes with mod
CNS -08	76.4	45.8	59.9	52.8	14.6	62.5	52.0	46.4	Yes with mod
CNS -09	80.6	47.2	58.9	63.9	16.7	64.6	55.3	53.6	Yes with mod
CNS -10	70.8	45.8	56.8	55.6	12.5	60.4	50.3	50.0	Yes with mod
CNS -11	75.0	45.8	59.9	63.9	14.6	62.5	53.6	50.0	Yes with mod
CNS -12	76.4	44.4	63.0	65.3	17.7	62.5	54.9	57.1	Yes
Mean (95% CI)	75.2 (72.8~77.6)	45.4 (44.8~45.9)	57.0 (55.7~58.3)	58.7 (56.4~61.0)	16.9 (14.3~19.4)	64.1 (62.3~65.9)	52.9 (52.2~53.5)	51.8 (51.0~52.6)	

**Figure 1:**
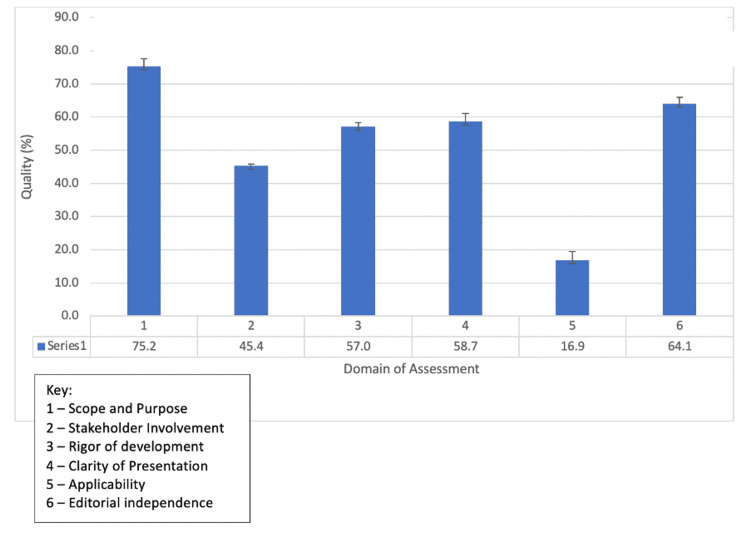
Assessment of mean quality of CNS guidelines based on AGREE II domain of assessment AGREE II: the Appraisal of Guidelines for Research and Evaluation II; CNS: the Congress of Neurological Surgeons

The mean domain score was highest for Scope and Purpose (75.2%), followed by Editorial Independence (64.1%), Clarity of Presentation (58.7%), and Rigor of Development (57.0%). The mean domain scores for Stakeholder Involvement (45.4%) and Applicability (16.9%) were deemed to be of the lowest quality (Figure 1).

Two domains exceeded the threshold for acceptable’ quality (60%), as described below

Scope and Purpose

Items 1-3: ‘The overall objective(s) of the guideline is (are) specifically described’, ‘the health question(s) covered by the guideline is (are) specifically described’, and ‘the population (patients, public, etc.) to whom the guideline is meant to apply is specifically described’.

Written information regarding these items was located in the Executive Summary document, which comprised CNS-02, and in additional paragraphs within the introductions of individual guidelines (CNS-03 to CNS-12).

Editorial Independence

Items 22-23: The guideline describes to what extent the ‘views of the funding body have not influenced the content of the guideline’ and the efficacy with which ‘competing interests of guideline development group members have been recorded and addressed’.

No guideline was considered unsuitable for recommendation by a majority of evaluators.

**Figure 2:**
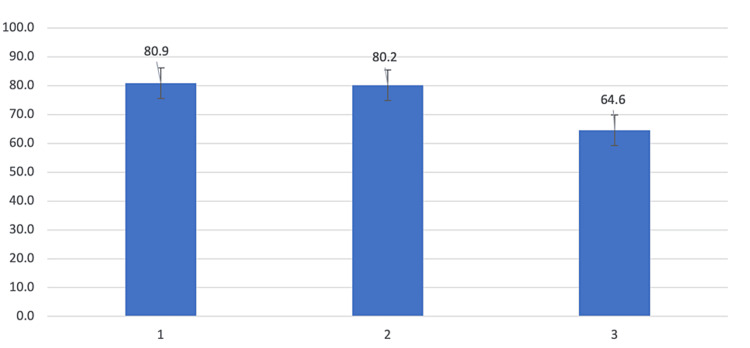
Assessment of item-specific scores for domain 1 (Scope and Purpose)

**Figure 3:**
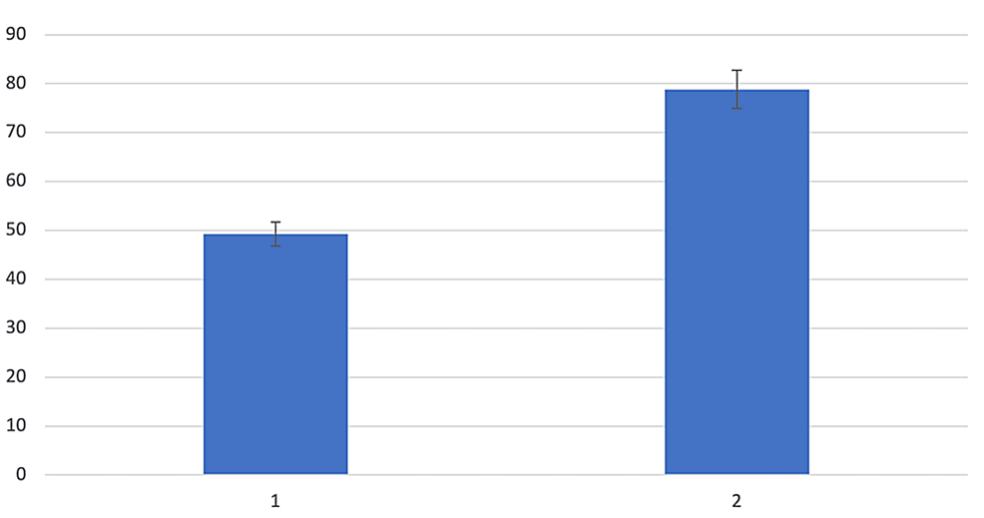
Assessment of item-specific scores for domain 6 (Editorial Independence)

For the domains that exceeded the acceptable quality threshold, a breakdown of each item-specific score is shown in Table 3 and illustrated in Figures 2-3.

**Table 3:** AGREE II instrument item-specific data of domains that exceeded the acceptable quality threshold (Items 1-3 and 22-23) for CNS guidelines on TST AGREE II: the Appraisal of Guidelines for Research and Evaluation II; CI: confidence interval; CNS: the Congress of Neurological Surgeons; TST: thoracolumbar spine trauma

Domain and domain item	Mean (95% CI)
Scope and Purpose	75.2 (72.8-77.6)
1. The overall objective(s) of the guideline is (are) specifically described	80.9 (79.6-82.2)
2. The health question(s) covered by the guideline are (are) specifically described	80.2 (79.2-81.2)
3. The population (patients, public, etc.) to whom the guideline is meant to apply is specifically described	64.6 (63.0-66.1)
Editorial Independence	64.1 (62.3-65.9)
22. The view of the funding body has not influenced the content of the guideline	49.3 (47.4-51.2)
23. Competing interests of guideline development group members have been recorded and addressed	78.8 (77.6-80.1)

Item-specific scores within Scope and Purpose revealed that specifying the population to whom these guidelines applied scored most poorly (64.6%), although this was still above the acceptable threshold. For the domain of Editorial Independence, the guidelines failed to achieve acceptable quality when specifically commenting on whether the views of the funding body influenced the content of the guideline.

Overall ICC for all CNS guidelines was 0.948 (average measures, two-way mixed effects model) (95% CI: 0.924-0.964), signifying excellent inter-rater reliability among appraisers. Individual ICC values for each CNS guideline ranged from 0.903 to 0.963 (Table 4), demonstrating excellent inter-rater reliability.

**Table 4:** ICCs for CNS TST guidelines CI: confidence interval; CNS: the Congress of Neurological Surgeons; ICC: intra-class correlation coefficient; TST: thoracolumbar spine trauma

	CNS-01	CNS-02	CNS-03	CNS-04	CNS-05	CNS-06	CNS-07	CNS-08	CNS-09	CNS-10	CNS-11	CNS-12
ICC	0.905	0.956	0.922	0.926	0.903	0.955	0.945	0.961	0.958	0.946	0.955	0.963
-95% CI	0.826	0.920	0.853	0.857	0.815	0.915	0.898	0.926	0.922	0.892	0.916	0.930
+95% CI	0.952	0.978	0.961	0.963	0.952	0.978	0.972	0.981	0.979	0.974	0.978	0.982

## Discussion

A recent analysis of Chinese orthopedic spine surgeons identified many barriers to the use of spine guidelines in their everyday work, including a lack of confidence in the content and applicability [16]. This study is designed to better inform spine surgeons about the strengths and limitations of their choices.

Although CNS guidelines for the management of TST generally demonstrated variable domain quality, on balance, our evaluators would recommend the guidelines to their peers with varying degrees of modification. Specifically, within the context of the "applicability" of CNS guidelines, we found that updated guidelines would benefit from a clear and robust evaluation of potential enablers and barriers to the application of CNS recommendations to wide-ranging hospital settings, including in resource-poor settings, and with associated cost estimations. Furthermore, future updates to CNS guidelines for the management of TST would benefit from seeking wider stakeholder involvement in the process of guideline development, particularly from the patient population to which the management guidelines would apply, and from allied healthcare professionals involved in injury rehabilitation.

TST injuries occur in around 7% of blunt trauma and account for 50-90% of traumatic spinal injuries in North America [1-5]. Coherent, clear, and high-quality guidelines to classify, assess, and manage patients with TST are therefore essential to optimizing outcomes in spinal injuries as a whole, and to ease the global health burden resulting from these injuries. However, there remains a lack of clear consensus on many aspects of care in TST patients, including classification and radiological evaluation, treatment of blood pressure dysregulation and associated spinal cord injury, nonoperative vs. operative management, and optimal operative standard of care [1-3,5,30-35]. Before these clinical guidelines were released by the AANS/CNS, no universally applicable and accepted evidence-based guidelines on the management of TST were available. The AANS/CNS should therefore be commended for their efforts to pursue these laudable aims through the release of the TST clinical practice guidelines.

This is the first study to evaluate the quality of the CNS guidelines on the management of patients with TST and it involved evaluation through a multidisciplinary international collaboration. As seen in our assessment, the guidelines showed domains of high quality and domains that would benefit from further optimization. The highest mean scores were scored for the domains Scope and Purpose (75.2%)”, and Editorial Independence (64.1%), both exceeding the threshold for "acceptable" quality (60%). The lowest scores were scored for Applicability (16.9%), Stakeholder involvement (45.4%), Rigor of Development (57.0%), and Clarity of Presentation (58.7%).

Reliability

ICC of overall guideline scores in our study fell between 0.903 and 0.963, reaffirming excellent inter-rater reliability [26]. The high ICC ratings observed not only reinforce the value of the AGREE II methodology as a reliable assessment tool for CPG but also suggest that AANS/CNS guidelines on TST can serve as a set of guidelines that can be reliably and fairly understood internationally, by medical professionals with a variety of sub-specialty interests and first languages.

Domains reaching the acceptability threshold

The highest mean scores were observed for the domains Scope and Purpose (75.2%)” and Editorial Independence (64.1%), both exceeding the threshold for "acceptable" quality (60%). Within Scope and Purpose, all items reached acceptable quality, with the highest scoring item being specific objectives of the guideline being clearly described. An overarching summary of information regarding the scope and aims of the guidelines was contained in a single document, which comprised CNS-02. Having this executive summary as a stand-alone separate document comes with both advantages and risks. The advantages include the fact that the information regarding the overarching themes and purpose of the guidelines produced is not duplicated, allowing for more relevant and focused information to be presented elsewhere. However, the disadvantages include the potential to introduce a lack of coherence between the summary document and the individual guidelines, which also contained individual sections on scope and purpose, particularly given there was no reference to the "executive summary" document in other CPGs. This technique of incorporating scope, purpose, and methodology in both a standalone document and in individual guidelines is similar to the North American Spine Society CPGs, which have a "technical report" that accompanies the CPG that details the scope, purpose, and methodology, in addition to paragraphs relating to this for each respective CPG [35].

The domains that scored most highly were consistent with other appraisals of CPGs within spinal pathology [22,36,37] and across other specialties [38,39]. Further updates could improve the consistency with which the population to whom the guideline is meant to apply is described. For example, a description of the regions of the spine that comprised the definition "thoracolumbar" for the guideline was well described, and the justification as to why these regions are taken together under the purview of "thoracolumbar", but separately to other regions of the spine, was comprehensively stated. However, whether these guidelines apply to all patients (including the adult and pediatric populations), or whether they apply to specific age groups, was not described. Furthermore, a clear paragraph or labeled table that describes the target population and user of each guideline may be useful in bringing the reader’s attention to this important aspect quickly and efficiently. Examples of CPGs that efficiently convey this aspect are the recent National Institute for Health and Care Excellence (NICE) Guidelines for Low Back Pain and Sciatica in patients over 16, where a short ‘Who is it for?’ section is explicitly described at the start of the guideline, and the North American Spine Society (NASS) CPG for the Diagnosis and Treatment of Lower Back Pain, where paragraphs are present at the start of the document with respective titles of "Intended User" and "Patient Population" [35,40].

Within Editorial Independence, the recording and accounting of competing interests in the guideline development group was the highest-scoring item (78.8%) and was performed thoroughly and consistently. Each guideline specifically included a paragraph on potential conflicts of interest, and the method by which conflicts were sought, evaluated, and reported was clearly described. The measures taken to minimize competing interests and a description of how conflicts influenced the development of guidelines were, however, less well reported and could be improved in further updates. There are specific guidelines and recommendations published by the Institute of Medicine (US) Committee on Conflict of Interest in Medical Research, Education, and Practice, which suggest methods by which competing interests can be minimized [41]. They suggest having a table detailing explicitly the policy for seeking and managing potential conflicts of interest included in CPGs, as has been previously presented by the American College of Chest Physicians [42].

Other recommendations to reduce the potential influence of conflicts of interest included ensuring a diverse expert panel from a variety of relevant professional and other backgrounds and having an explicit and systematic method for reviewing relevant evidence. The most important recommendation involved using systematic procedures to evaluate the evidence and explicitly linking recommendations to the evidence. An explicit link between evidence and recommendations was not present in the CNS CPGs; however, this has been well executed in other CPGs of spine pathology such as the Danish Health Authority’s National Clinical Guidelines for non-surgical treatment of patients with recent-onset low back pain or lumbar radiculopathy [43]. Here, each recommendation was accompanied by a grade for the quality of evidence associated, and the specific studies that were considered in the recommendation were explicitly referenced.

Overall, across the CNS guidelines on TST, the ability to report that "the views of the funding body have not influenced the content of the guideline" scored less well (49.3%). Encouragingly, each guideline stated funding information under a separate paragraph entitled "Disclosures", and clearly stated that guidelines were funded internally by the CNS and the "Section on Neurotrauma and Critical Care", which contributed to domain evaluation. However, there was no statement that the funding body did not influence the content of the guideline, which is an essential aspect of this declaration. Further updates of these guidelines may require a more explicit declaration of whether the funding body influenced the content of guidelines, and if so, how this may influence the guidelines and how this may be mitigated, as per the recommendations of the Institute of Medicine (US) Committee on Conflict of Interest in Medical Research, Education, and Practice [41].

Although failing to exceed the acceptable quality threshold, the domains Clarity of Presentation (58.7%) and Rigor of Development (57.0%) achieved scores close to those deemed acceptable quality and may require only minor modifications in further updates. The domain Rigor of Development evaluates the methodology employed to synthesize the evidence and formulate recommendations [26]; a planned update process is also required [44]. Within this domain, aspects that could be improved were clear descriptions of the methodology for formulating the recommendations and including an external review of the guideline. For example, within the methodology, a clear summary of the inclusion and exclusion criteria for each guideline could be incorporated in further updates. Furthermore, the authors could not find a scheduled date being provided publicly for updating the CNS guidelines, and this could be rectified. The systematic search for evidence could be more explicitly described by stating the Boolean terms used for the systematic search, and including reasons for exclusion of full texts within the PRISMA flowchart.

A recent evaluation of the North American Spine Society guidelines using the AGREE II instrument identified this domain to be one of the guideline’s highest-scoring domains due to its transparent and explicit descriptions of the search strategy and selection criteria in this regard [18]. Modification of these aspects of this domain would substantially strengthen further guidelines within TST. Ensuring a high score for Rigor of Development within a CPG is important, as the quality of this domain has been identified as a strong predictor of quality by the AGREE instrument [45]. Hoffman-Esser et al. [45] have previously shown through regression analysis that there is a statistically significant influence on the assessment of the items in this domain and overall guideline quality.

Domains not reaching the acceptability threshold

Mean domain scores for Stakeholder Involvement (45.4%) and Applicability (16.9%) were of low quality and thus may require significant modification in further guideline updates. Our findings are in line with previous appraisals of CPGs across several contexts within spine pathology and other specialties, which suggest room for improvement within these domains [46-49].

As with other CPGs [21,27,37,38,45], the domain Applicability achieved the lowest scores, ranging from 12.5 to 21.9. There appears to be a consistent trend for CPGs to score poorly in this domain, with recent appraisals of other spinal guidelines reporting scores ranging from 13 to 21% [21,27] and 0-86.5% [37]. The marked lack of implementation advice across multiple CPGs from a wide variety of specialties has been noted in a recent systematic review [48]. Although CPGs can provide healthcare professionals with the necessary guidance to access the best research evidence efficiently, without efficient implementation advice, CPGs can have a limited effect on changing clinical behavior. Indeed, no guideline in our assessment robustly evaluated the facilitators and barriers to its application or suggested efficient methods of implementation.

Applicability of guidelines is crucial to their successful adoption, but is often dependent on the infrastructure and available resources within health systems, and therefore requires specific consideration. Implementation tools such as an audit tool with an extractable spreadsheet to assist clinicians in objectively assessing the impact of new or updated guidelines may be worthwhile in improving the applicability and implementation of these CPGs. However, it has been noted that multiple factors influence CPG applicability, such as patient, provider, institution, and system-level issues, indicating why this domain consistently scores most poorly across CPGs [48]. It may be worthwhile for future updates to dedicate a specific document or paragraph within individual guidelines to evaluate this domain and combine this with an audit tool to assess implementation.

Stakeholder Involvement involves including individuals from all relevant professional groups when developing the guideline, actively seeking out and incorporating the views and preferences of the target population, and identifying the target users of the guideline [26]. Within this domain, the specific aspect of seeking out the views and preferences of the target population consistently scored poorly. Indeed, no guideline within our evaluation explicitly commented on how they included the views and preferences of patients. Again, this has been previously reported in other appraisals of CPGs within spine pathology [27,37]. A formal procedure for early involvement of relevant patient populations, with methods to advertise and encourage patient involvement may be required to broaden patient involvement in guideline production across all disciplines. Online surveys and social media platforms may prove to be important tools in encouraging patient involvement in this way, but further evaluation of these techniques is required.

Limitations

This study has a few limitations. Firstly, AGREE II is a subjective evaluation tool and thus is susceptible to bias. Second, our evaluation comprised five independent appraisers, and it is likely that a greater number of appraisers may provide a more robust evaluation of the CNS guidelines of TST. However, the AGREE II working group has previously described that the ICC provided by four appraisers is better than three or two, and thus including five is likely to lead to a robust method of evaluation given the practical limitations of CPG quality assessment [26]. Lastly, the scope of our study was limited to appraising one widely used set of guidelines from a single national body based in the United States, CNS. Guidelines from other national and international bodies were not subject to this appraisal. However, the authors are unaware of other widely published national guidelines specifically aimed at the management of TST.

## Conclusions

Although widely used, the CNS guidelines on TST have not yet been subject to external appraisal using a validated tool and procedure. This study involved the first external independent appraisal of TST guidelines in the literature, using a validated tool. Our consensus is that while CNS guidelines for the management of TST generally demonstrated an overall acceptable quality, individual domains could benefit from modification in subsequent updates, specifically the domain Applicability. The pattern of domains that performed strongly and those that performed weakly are largely consistent with other appraisals performed of other CPGs in the literature, both within and outside of spine disease. Domains scoring highly in this appraisal should be considered and referenced as exemplars of good guideline practice by future developers. We suggest that an updated evaluation of CNS guidelines for the management of TST and related disorders is required, which incorporates the recommendations discussed to optimize robust and effective CPGs and can be implemented efficiently.
